# Functional and patient-reported outcomes of the Swanson metacarpo-phalangeal arthroplasty in the rheumatoid hand

**DOI:** 10.1007/s00402-017-2675-1

**Published:** 2017-03-24

**Authors:** Firas K. Elherik, Sean Dolan, John Antrum, Frank Unglaub, Colin R. Howie, Steffen J. Breusch

**Affiliations:** 1 Department of Orthopaedic Surgery, New Royal Infirmary of Edinburgh, Little France, Edinburgh, EH16 4SU UK; 2 University of Dundee Medical School, Ninewells Hospital, Dundee, DD2 1UB UK; 3 Department of Hand Surgery, Vulpiusklinik, Bad Rappenau, Germany

**Keywords:** Swanson, Metacarpo-phalangeal joint, Silastic implants, M-SACRAH, Patient-reported outcome measures, Rheumatoid arthritis

## Abstract

**Introduction:**

Replacement of the metacarpo-phalangeal joints (MCPJ) with silastic Swanson’s implants can help decrease pain, stiffness and allow for improved function in rheumatoid arthritis (RA). There is a lack of patient reported outcome measure (PROM) studies assessing the efficacy of this procedure in RA. The aim of this study was to report any change in function, pain, stiffness and satisfaction following the Swanson MCPJ replacement using patient reported outcomes in a rheumatoid population.

**Methods:**

The combined results of 64 RA patients (71 hands) with 284 Swanson MCPJ arthroplasties (mean follow-up: 75.85 months) were assessed using the validated M-SACRAH questionnaire and a separate satisfaction questionnaire. Radiographic evaluation was performed to insure correct alignment of the hinged prosthesis postoperatively. No attempt was made to identify other predictors, radiologically or clinically. Data analysed in the study was interpreted in the context of the number of hands and survivorship was defined as implant fracture, loosening or revision.

**Results:**

The mean total functional outcome score improved by 46.2% and the total pain outcome improved by 60.2%. The total stiffness outcome improved by 56.9% postoperatively and the results obtained from the satisfaction questions revealed that 73.2% of patients would retrospectively elect to have the procedure again. We report two postoperative complications in this group of superficial wound infections. Radiographically, all MCPJs showed improved alignment, however five patients reported worsening pain, four patients reported increased stiffness and four reported reduced function postoperatively. There was one re-operation of a 5th MCPJ Swanson’s, which did not require implant exchange and one implant was revised. Implant survivorship was 98.6%.

**Conclusions:**

Patient satisfaction and functional surrogate markers were overall favourable. Our results support the continued use of Swanson silastic arthoplasty in advanced RA.

## Introduction

**Fig. 1:**
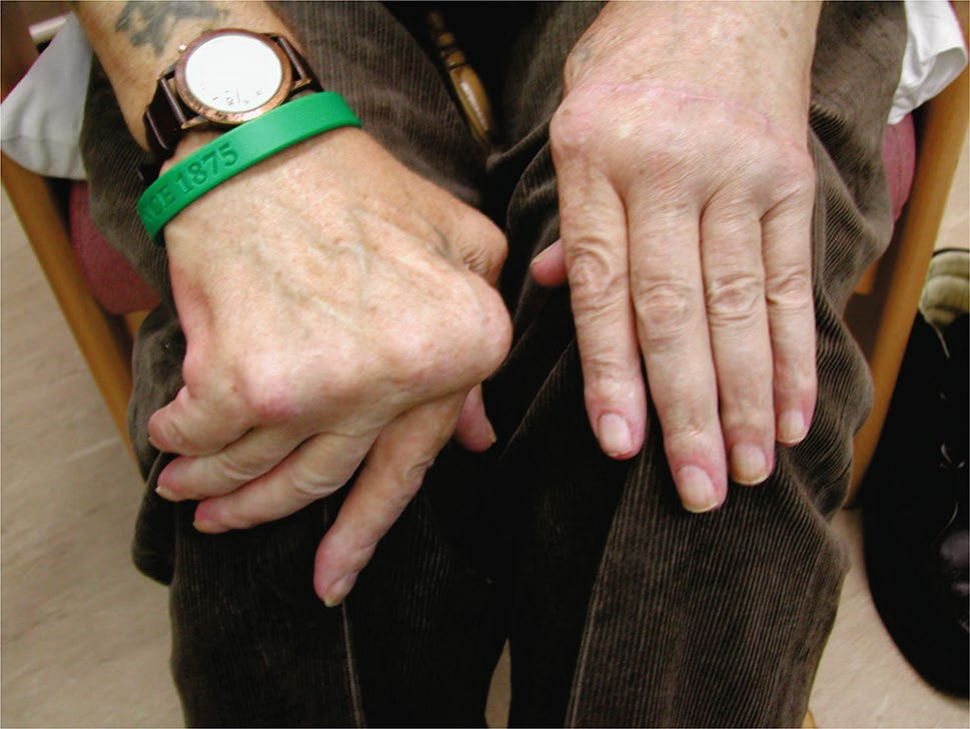
The end stage rheumatoid MCPJ is classically subluxed or dislocated volarly, fixed in flexion with ulnar deviation of the fingers. *Left hand* 4 years postpostoperatively, *right hand* prior surgery

It is estimated that approximately 600,000 people in the United Kingdom are affected by rheumatoid arthritis. A prevalence study reports that the disease effects 1% of men and 3% of women worldwide [1]. Rheumatoid arthritis is a symmetrical inflammatory poly-arthropathy characterised by progressive cell-mediated destruction of the joints, with early predisposition for the small joints of the hands and feet. The metacarpophalangeal joint (MCPJ) is the most commonly involved joint in the hand and is seen early in the disease process [2]. Although the aetiology of the deformity is not fully understood, a proliferative and destructive synovitis of the articular surfaces with a loss of supporting structures of the joint is thought to be the driving mechanism of deformity. The end stage rheumatoid MCPJ is classically subluxed or dislocated volarly, fixed in flexion with ulnar deviation of the fingers (Fig. 1) [3]. Unfortunately, however RA is not limited to the MCPJ articulation and the classic wrist collapse leads to radial deviation of the metacarpals, further exaggerating the deforming forces acting upon the MCPJ [3]. As a result of these processes patients can develop intractable pain, reduced function and progressive deformity incompatible with daily living. The National Institute for Health and Clinical Excellence (NICE) guidelines emphasise the importance of early introduction of disease modifying anti-rheumatic drugs (DMARD), often combined with the use of biological therapies [4]. Though becoming less common with early aggressive medical management, surgical intervention is indicated when there is persistent pain, synovitis, worsening function or progressive deformity despite optimal medical therapy [5]. Replacement of the MCPJ is a well-established procedure and the most widely used implant utilises silastic flexible hinge prosthesis [6].

Silicone implants have been used since the late 1960s to replace destroyed MCPJs in the rheumatoid hand. Swanson first described MCPJ replacement using a hinged, double stemmed silicone implants in 1969. This has emerged as the preferred, most commonly used implant because of its durability and flexibility [7]. The most common indication for Swanson’s arthoplasty of the MCPJ in RA is pain and loss of function in the late stage deformity [6, 8]. Other implants which can be used include the unconstrained two piece pyrolytic carbon implants but are not regularly used due to expense and concern with their ability to resist flexion and ulnar deviation forces in comparsion with their silicone counterparts [3].

Although radiological and physical outcomes are clearly important in assessing biomechanical changes [2] they have limited bearing unless they are considered in context of the patients perceived benefit in terms of reducing pain, stiffness, improving function and ultimately quality of life. However, relatively little is known in relation to patient-reported outcomes after Swanson’s MCPJ replacement. The objectives of this study were to report any change in patient reported outcomes of function, stiffness and pain following the Swanson MCPJ arthroplasty in a two senior surgeon series of rheumatoid patients using the validated Modified-score for the assessment and quantification of chronic affections of the hand (M-SACRAH) questionnaire [9]. A separate questionnaire was used to assess general satisfaction following the procedure. We are aware of only one other smaller prospective outcomes study assessing efficacy of this procedure [10]. The intent of this report was not to necessarily evaluate the Swanson implant per se, but to evaluate the effectiveness from the patient’s perspective which could be used to provide useful quantitative data and information for patient counselling.

## Materials and methods

From our local arthroplasty database of two senior surgeons over an 18 year period, 64 patients (71 hands) with long standing RA who had undergone the Swanson 2–5 MCPJ arthroplasty (284 implants) were identified. The mean follow-up period was 75.85 months (range 3–207) months. One patient died from unrelated causes to the procedure and was lost to follow-up. All arthroplasties were performed in the same hospital by one of two senior consultant surgeons (SJB, CRH) and routinely on the end stage rheumatoid hand (Larsen grade 4 or 5) using the same surgical technique (though slightly modified) and postoperative management as described by Swanson [6]. The primary outcome measures were the same for all patients and included the validated M-SACRAH score and a separate departmental satisfaction questionnaire. The M-SACRAH is a validated 12-point questionnaire assessing the rheumatoid hand in three domains: (1) hand function (eight questions):‘possible without any difficulty’ (0) to ‘impossible’ (100); (2) stiffness (two questions), using a range from ‘no stiffness’ (0) to ‘unbearable stiffness’ (100); (3) pain (two questions), using a range from ‘no pain’ (0) to ‘unbearable pain’ (100).

After gaining verbal consent, patients were asked to complete the M-SACRAH questionnaire assessing preoperative and postoperative level of pain, stiffness and function. This study was largely prospective in its data collection, but some data had to be collected retrospectively. Patients operated on or after 2010 had their preoperative questionnaires collected prospectively at preassessment clinic using the M-SACRAH. Prior to 2010 the author’s own prospective data recording in patient medical notes provided the data for the preoperative M-SACRAH questions. Any outstanding (10 out of 12 questions; function 1/8 locking/unlocking a door and 8/8 writing by hand) questions hence had to be obtained in a retrospective manner to allow comparative M-SACRAH scoring.

In addition, patients were asked to answer three individual satisfaction related questions: (1) overall how pleased have you been with the results of your surgery so far? (Very pleased, fairly pleased, not very pleased, very disappointed), (2) in hindsight, would you still elect to have the procedure done? (Yes/Maybe/No), (3) would you recommend this procedure? (Yes/Maybe/No). Radiographic evaluation was performed to insure correct alignment of the hinged prosthesis postoperatively. No attempt was made to identify other predictors, radiologically or clinically and data analysed in the study was interpreted in the context of the number of hands. The aim of this study was to assess the success and overall satisfaction following the Swanson MCP arthroplasty using patient reported outcomes. Postoperative complications including infection, re-operation, stiffness as well as implant survival rate were determined. Survivorship was defined as implant fracture, loosening or revision. Statistical comparisons were performed in Excel using two-sided paired Student’s *t* test examining outcomes before and after surgery. Differences were deemed statistically significant if the *p*-value was <0.05.

### Surgical technique

**Fig. 2:**
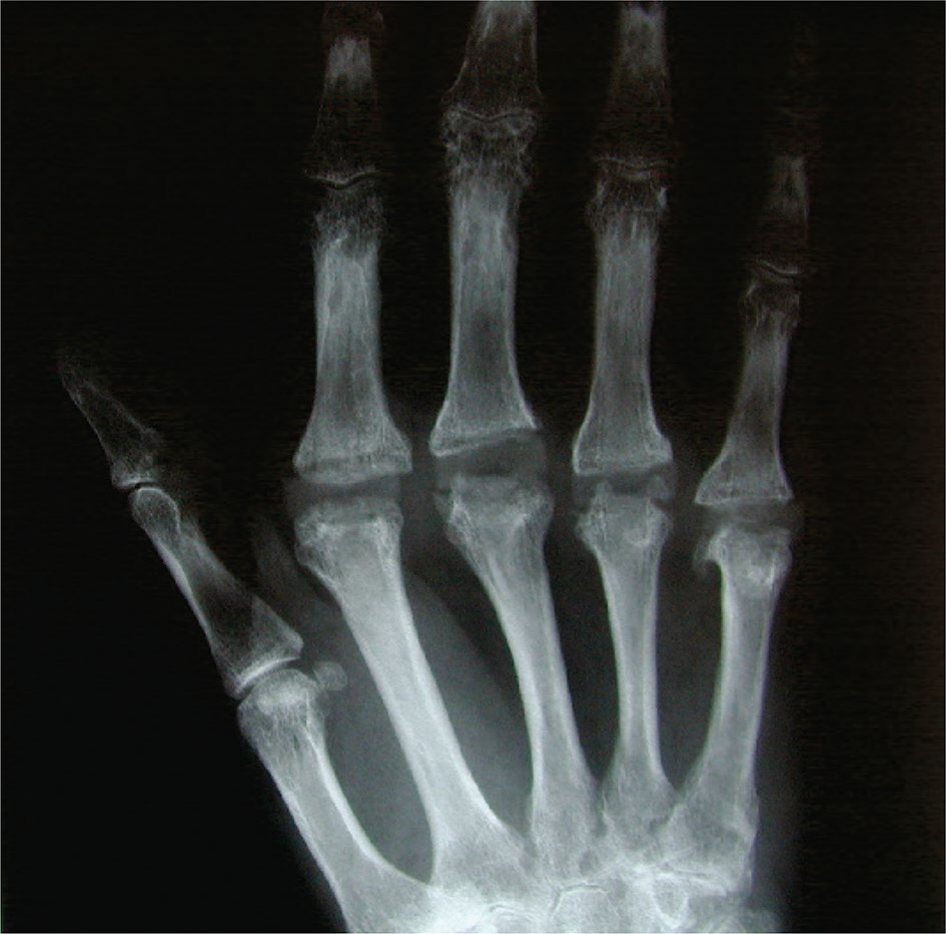
The postoperative radiographic appearance of the Swanson silastic MCPJ arthroplasty. Radiographs show no evidence for implant failure

All procedures were performed by the senior surgeons (CRH or SJB) in accordance (although albeit slightly modified) with the technique described by Swanson [6]. General anaesthetic and nerve block, 1.5 g IV Cefuroxime at induction, above elbow tourniquet. A transverse dorsal skin incision was made from 2nd to 5th MC heads to allow access to all MCPJs. The dislocated extensor hood was released laterally with release of paratendon along the full length of the proximal phalanx extending proximally to the MCs. The extensor tendon was then retracted medially to expose the joint for synovectomy, release of the ulnar collateral ligaments at the 5th digit and release of abductor digiti minimi. Full synovectomy, exposure and sharp capsular release of the metatarsal head and base of proximal phalanx was required to allow correction of volar flexion and ulnar deviation contractures. After resection of the MC head a full capsular release was then possible. After osteotomy of the proximal phalanx base both the MC and phalanx intramedullary cavities were reamed and prepared using small hand broaches to ensure tight canal fit of the trial implants. The largest possible implant was used and reefing of radial collateral with trans-osseous non-absorbable Ethibond sutures was done. If necessary further Vicryl reefing sutures were used to ensure relocation and fixation of the extensor tendons. Washout, haemostasis, skin closure and tourniquet released. Gauze dressing, cotton wool bandage and crepe bandage. Volar slab with hand in functional position. All patients routinely had X-rays and cefuroxime IV postoperatively. Wound inspection at day 4–6 postoperatively at first physiotherapy appointment as per local integrated care pathway. The postoperative physiotherapy programme included night splinting and regular exercise session for up to 3 months [11]. Figure 2 demonstrates the postoperative radiographic appearance of the Swanson silastic MCPJ arthroplasty.

## Results

One patient had died at the time of follow-up from causes unrelated to the surgery. The baseline characteristics are outlined in Table 1.

**Table 1:** Baseline characteristics of the study population

Demographics	*n* = 71
Gender	Female (60), male (11)
Age (years); mean (range)	63.2 (42–83)
Procedure on dominant hand	44 (62%)
Follow-up (months) mean (range)	75.85 (2-207)
Mean disease duration (range)	9.13 years (2–12)
Disease modifying therapies (%)	Methotrexate (42.2%), None (23.9%), Etanercept (7%), Hydroxychloroquine (9.9%), Azathioprine (7%), Sulfasalazine (8.4%) Leflunomide (8.4%) Rituximab (4.2%)
Polyarticular disease	58 (81.7%)

## Outcomes

Highly significant improvements in all domains of function, pain and stiffness scores were reported following the Swanson MCPJ arthroplasty. The majority of patients reported improvement in all areas, however individual variation in response to surgery was observed with five patients reporting moderately worsening of pain, four patients reporting a deteriorating function due to stiffness and four patients reported worsening stiffness postoperatively. One patient reporting no change in stiffness, however this did not correlate with deteriorating function. Overall there is a remarkable uniformity in the observed reported outcomes. Summary of the results for all interventions is reported in Table 2. In keeping with these positive results very high levels of overall satisfaction were observed with 73.2% of patients reporting that they would retrospectively elect to have the procedure again and 84.5% would recommend the procedure, with the majority of patients being “very pleased” with the outcome of their surgery (58% “very pleased”, 28.2% “fairly pleased” and 14% “dissatisfied”).

**Table 2:** Function, stiffness and pain outcomes of the M-SACRAH questionnaire

	Preoperative mean (range)	Postoperative mean (range)	% Improvement	*P* value
Function				
Locking/unlocking a door	78.6 (10–100)	42.1 (0–100)	46.4	<0.001
Buttoning up/unbuttoning shirt/blouse	84.5 (10–100)	46.3 (0–100)	45.2	<0.001
Turning a water tap	80.4 (20–100)	43.1 (0–100)	46.4	<0.001
Fastening/unfastening a zip	81.9 (20–100)	45.9 (0–100)	43.9	<0.01
Tying shoelaces	85.5 (40–100)	45.3 (0–100)	47	<0.001
Unscrewing a toothpaste cap	80.4 (20–100)	43.2 (0–100)	46.3	<0.001
Turning the pages of a newspaper	62.95 (0–100)	30.8 (0–90)	51.1	<0.001
Writing by hand	78.45 (10–100)	43.5 (10–100)	44.5	<0.001
Total	**632.8 (300–800)**	**340.4 (90–780)**	**46.2**	<0.001
Stiffness				
Morning stiffness	83.7 (30–100)	34.64 (0–100)	58.6	<0.001
Stiffness later in day following inactivity	75.35 (20–100)	31.8 (0–100)	57.8	<0.001
Total	**159 (50–200)**	**68.5 (0**–**200)**	**56.9**	<0.001
Pain				
Pain during intensive work	81.3 (10–100)	36.1 (0–100)	55.6	<0.001
Pain at times of inactivity	72.2 (20–100)	25.1 (0–90)	65.2	<0.001
Total	**153.5 (50–200)**	**61.1 (0**–**180)**	**60.2**	<0.001

Bold values indicate statistically significant

Consistent and highly significant improvements across all outcomes are reported following the Swanson MCPJ arthroplasty in all parameters assessed. *P* values represent the result of paired Student’s *t* test comparing pre- and postoperative scores

### Complications and radiographs

We report two postoperative complications in this group of superficial wound infections in patients on Methotrexate, which resolved with a course of oral antibiotics. There was one re-operation of a 5th MCP Swanson’s due to delicate soft tissue coverage and worry of skin breakdown. At re-operation the Swanson was not exchanged but the Silicon ridge laterally was trimmed with a knife. One implant was revised at 9 years due to clinically evident silicon synovitis. Radiographically, all MCPJs showed good anatomical alignment at the scheduled 1 year follow-up with no significant recurrent ulnar deviation (>10°). Thirty-three patients did not have up to date radiographs for assessment. There was radiographic evidence confirming three implant fractures (4.22%), however these did not require revision as there was no clinical evidence of silicone synovitis.

## Discussion

The Swanson’s arthoplasty technique offers excellent immediate results with reduction in pain, improvement of arc of motion and correction of the deformity [12]. Despite the long term use of the Swanson implants, the current evidence-based literature assessing this procedure in RA is limited, consisting mostly of retrospective case series that are predominantly based on radiological and physical outcomes such as grip strength and range of motion or report on other patient groups [13]. The limited long-term studies that have been published demonstrate a high rate of implant failure due to fracture (up to 58%) or loosening of the silastic implants [13, 14]. The definition of loosening is obscure as these silicone implants are by definition never firmly fixed or ingrown. The main failure mechanisms are fracture and silicone wear leading to synovitis, both of which we encountered in our series. The implant fracture rate was apparently low, but some implant fractures may have been missed, as 33/64 patients had no up to date radiographs at time of follow-up, which is the main limitation of our study. Despite this radiographic failure; Swanson’s MCPJ arthroplasty has remained a popular procedure [2, 3, 5, 6]. There is however limited information available regarding the long-term clinical outcome of Swanson MCPJ in this patient group with only one study assessing the long-term outcome of silicone MCPJ, which did not report exclusively on the Swanson prosthesis [15].

Although patient-reported outcome measure (PROM) studies are becoming more fashionable in assessing the effectiveness of surgical procedures, we are aware of only one other study in the published literature that reports on the effectiveness of this procedure using PROM’s. Our results compare well with other studies reporting favourable results in the short term [6, 16–18]. The fact that some of the data for the validated questionnaire had to be obtained retrospectively has to be regarded as a significant limitation of our study, but we still feel that as the majority of data was collected in a prospective manner, that our findings are of clinical value. Patient expectations of MCP joint arthroplasty are high [19] and our results may be used by rheumatologists and surgeons to provide better patient counselling and help set realistic expectations. A study of 33 patients with RA who received silicone MCPJ arthroplasty concluded that the greatest motivation for surgery was functional improvement with pain reduction ranked second. In addition to this they reported that aesthetic appearance should also be considered an important motivation for surgery and determinant of satisfaction in MCP joint arthroplasty in RA [19]. A prospective study of 137 patients compared the expectations and outcomes of function and pain for surgical (silicon MCPJ arthroplasty) and non-surgical (medically managed) RA patients over an average follow-up period of 6.7 years. Their report highlighted that although patients tended to be over-optimistic about the outcome of surgery; overall a higher proportion of surgical patients were more satisfied with their treatment and had greater levels of hand function at long-term follow-up [20].

An additional important factor in hand biomechanics that makes silicon MCPJ challenging is that it is difficult to evaluate each joint in isolation because there is substantial interplay between adjacent joints in the hand with some advocating evaluation of surrounding joints before planning silicon MCPJ arthroplasty [21]. Two of the more common hand deformities in RA are swan neck and boutonniere deformities. It had been postulated in the past that boutonniere deformity as having a detrimental effect on MCPJ arc of motion thus requiring correction prior to MCPJ arthroplasty. In contrast, proximal interphalangeal joint hyperextension in the swan neck deformity creating greater need for metacarpophalangeal joint motion to make a fist thus, recommending delaying the treatment after silicone metacarpophalangeal joint arthroplasty. One group tested this hypothesis by assessing the arc of motion in 73 rheumatoid MCP joints as well as functional hand outcomes using the Michigan hand outcomes questionnaire. Their study did not support the theory that boutonniere deformity has negative effects on MCPJ arc of motion despite having worse baseline function. They also reported that RA patients with swan neck deformities did not have a greater MCPJ arc of motion before or after surgery, thus concluding that MCPJ arthroplasty should be performed before treatment of swan neck or boutonniere deformities [22].

In the cohort analysed in this study, patients reported an overall decrease in pain with the majority (58%) very pleased and most (84.5%) would retrospectively have the procedure performed again. These favourable satisfaction outcomes have been echoed by other studies in the published literature [23, 24]. Patient pain and functional surrogate markers were also overall very favourable. Noticeable individual variation in response to surgery was observed with four patients in this group reporting a substantial loss of function postoperatively and five patients reporting moderately worsening of pain following their procedure. It is important to note that all these patients had longer disease duration, severe baseline polyarticuar disease (90% Larsen grade 5) and longer follow-up period compared to other patients in this group. Therefore the inferior results at longer term follow-up may merely represent gradual deteriotion over time with ageing and RA progression. A longituninal study with regular follow-up interval recordings would be required to determine this relationship and this has now been implemented in the one of the authors prospective sub-cohort of patients. Despite this apparent functional loss however; it has been demonstrated that patient satisfaction improves after silicone MCPJ arthroplasty despite minimal change in hand function. One prospective, multicentre study that examined the intersect between functional outcomes and patient satisfaction concluded that clinically successful MCPJ replacement does not need to fully restore the full ROM of the joint in the rheumatoid hand. In their report MCPJ arthroplasty did not increase ROM however this was not associated with any adverse satisfaction outcomes [25].

Despite the limitations in observational studies of this kind we still feel that overall there is a remarkable uniformity in the observed reported outcomes suggesting the Swanson MCPJ replacement to be an effective procedure in this patient population with mainly favourable results in terms of reduction in stiffness, pain, improvements in function and good satisfaction results. In our opinion therefore this procedure should still be considered as a viable option in management of rheumatoid patients with intractable pain and progressive loss of hand function.
